# Should We Ever Pace for Carotid Sinus Syndrome?

**DOI:** 10.3389/fcvm.2020.00044

**Published:** 2020-04-22

**Authors:** Steve W. Parry

**Affiliations:** Newcastle University Institute of Ageing, Newcastle University, Newcastle upon Tyne, United Kingdom

**Keywords:** pacemaker, carotid sinus syncope, neurally mediate syncope, syncope - etiology, treatment - contemporary views

## Abstract

Carotid sinus syndrome has been associated with transient loss of consciousness for millennia, and while steeped in cardiovascular lore, there is little in the way of solid evidence to guide its main treatment modality, permanent cardiac pacing. This article reviews the history of the condition in the context of its contemporary understanding before examining three key concepts in the consideration of what constitutes a manageable disease: first, is there a pathophysiologic rationale for the disease (in this case carotid sinus syndrome)? Second, is there a good diagnostic test that will identify it reliably? And finally, is there a convincingly evidence-based treatment for the disease? Relevant literature is reviewed, and recommendations made in how we view pacing in the context of this intriguingly opaque condition.

## Historical Context

**Figure 1:**
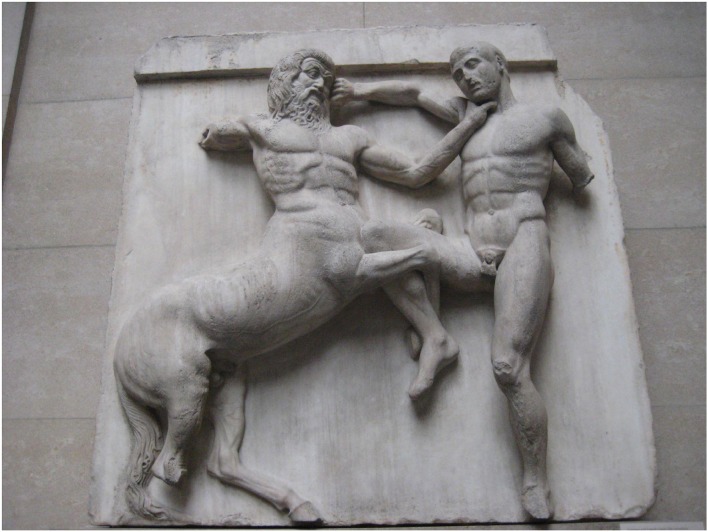
Centaur and Lapith, 31st Metope, The Parthenon. By Claire H., originally posted to Flickr as Centaur and Lapith, CC BY-SA 2.0 (https://commons.wikimedia.org/w/index.php?curid=5123552).

**Figure 2:**
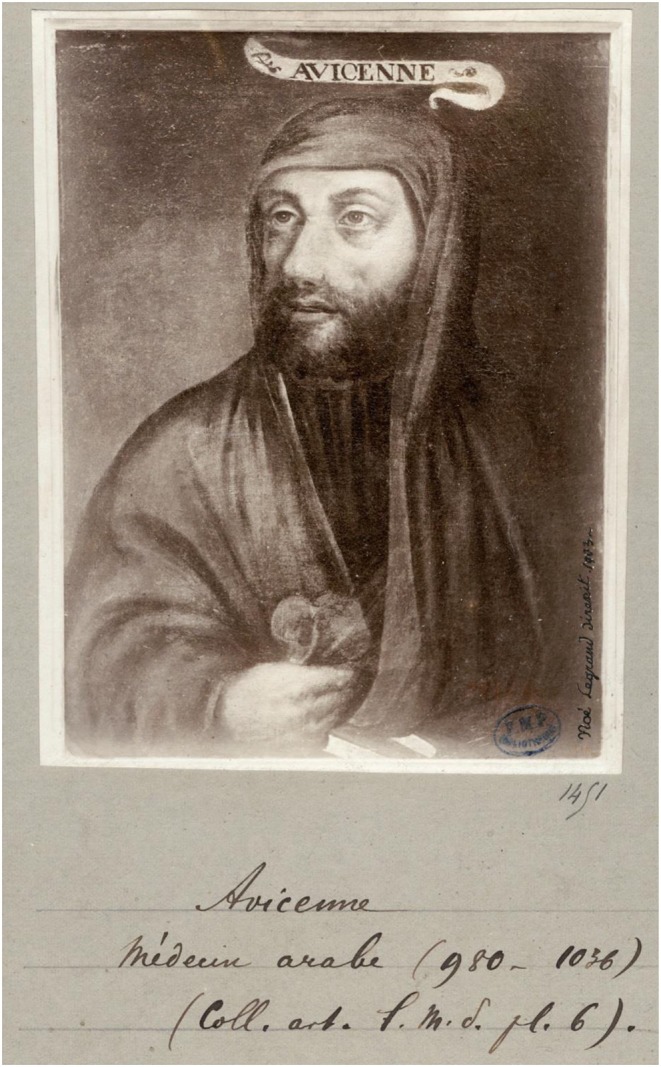
Avicenna (Ibn Sina). From Wikimedia Commons, *via* Bibliotheque Interuniversitaire (https://www.commons.wikimedia.org/wiki/Category:Media_contributed_by_the_Biblioth%C3%A8que_interuniversitaire_de_sant%C3%A9).

The association of the carotid sinus with impaired consciousness stretches back over several millennia. The ancient Assyrians used carotid compression to dull the pain associated with ritual circumcision (1), while one of the Parthenon's metopes from the 5th century BC illustrates the offensive use of carotid compression by a centaur to cause unconsciousness in an opposing Lapith soldier (Figure 1). Six centuries later, the ancient Greek physician and philosopher Galen (AD c 130–210) wrote of the loss of consciousness caused by the compression of nerves surrounding the carotid arteries (2), while the Greek recognition of the physiologic significance of the carotids is evident, the name being derived from the Greek *karotides*, the plural of *karotis*, meaning drowsiness, which itself was derived from the verb *karoun* (to stupefy). The Persian Muslim father of modern medicine Avicenna (c 980–1037; Figure 2) later commented on falling and unconsciousness induced by carotid sinus pressure by hammams in public baths (3, 4), while the French barber surgeon to several kings Ambroise Paré (c 1510–1590) noted that “(the) two branches which they call carotides or soporales, the sleepy arteries, because they being obstructed, or any way stopt we presently fall asleep” (5).

**Figure 3:**
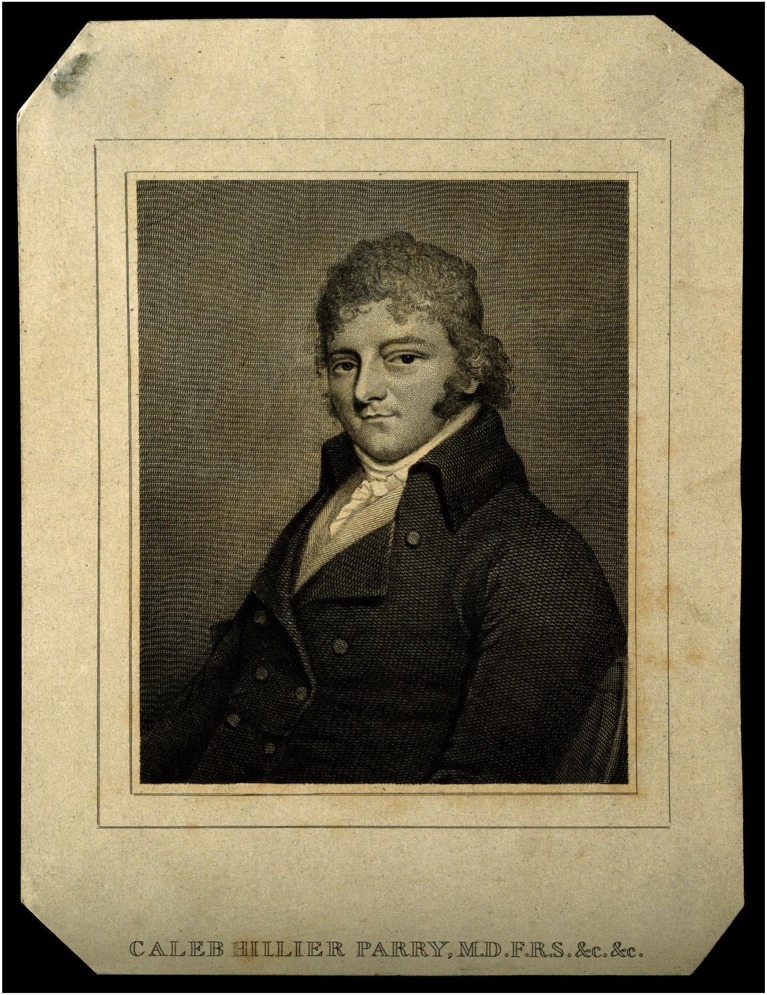
Caleb Hillier Parry. Engraving by P. Audinet after J. H. Bel; from Welcome Images, *via* Wikimedia Commons (https://commons.wikimedia.org/wiki/File:Caleb_Hillier_Parry._Engraving_by_P._Audinet_after_J._H._Bel_Wellcome_V0004501.jpg, original at http://catalogue.wellcomelibrary.org/record=b1171293).

However, it was not until 1799 that the English physician and friend of Edward Jenner (initiator of the smallpox vaccine), Caleb Hillier Parry (Figure 3), made the more causal observation between carotid pressure and syncope, noting that “in patients, whose hearts have been beating with undue quickness and force, I have often, in a few seconds, retarded their motion many pulsations in a minute, by strong pressure on one of the carotid arteries,” though he took this to be a sign of coronary artery disease (6). In 1862, further observations were recorded by Waller on the effect of pressure over the carotid artery posterior to the ramus of the mandible: “The heart beat at first increases in number with decreased power followed by a retardation of the heart action of about four to five beats a minute….,” an action he attributed to vagal activation (6).

Waller noted with great insight that “it is easily ascertained that the symptoms above described are not owing to *compression* of the carotid artery, as they may be produced without obliteration of the calibre of the artery; or vice versa the course of the blood may be completely interrupted in the artery without producing any of the symptoms enumerated,” (7) providing an early distinction between the pathologic and the physiologic reflexive hemodynamic changes and the importance of symptoms in attributing causation in the clinical setting. Four years later, these observations were expanded upon by the Austrian physiologist Czermak (8), who found that self-induced carotid pressure at the level of the upper margin of the sternocleidomastoid muscle caused temporary slowing of the heart rate, which was more pronounced on the right than on the left. Czermak's conclusions regarding the mechanism of cardiac slowing attributed to vagal pressure in the region of the carotid sinus held sway for much of the ensuing 50 years, with the test itself denoted as the “vagus druckversuch” or “vagus pressure test” (9).

In 1912, Sollman and Brown showed that traction on the carotid arteries caused a relative bradycardia and fall in blood pressure independent of vagal stimulation (10), but it was not until a decade later that Hering showed that mechanical pressure at the bifurcation of the common carotid artery caused cardioinhibition, even when the vagus was dissociated from the artery (11, 12). Hering's associate Koch (13, 14) confirmed these observations, while de Castro (15) and Heymans (16) showed that the carotid sinus was richly supplied with sensory receptors (15, 16) found predominantly in its adventia, emerging as spiral fibers which unite to form the carotid sinus nerve, Hering's nerve (12), or the intercarotid nerve of de Castro (15). Sunder-Plassman (17) later showed the union of the carotid sinus nerve with the hypoglossal nerve, conclusively demonstrating the direct afferent connection between the carotid sinus and the brainstem.

Clinical studies on the physiology and the pathophysiology of the carotid sinus in human subjects only really began following the discovery of a reflexive role for the sinus independent of the vagus nerve. Koch (13) studied the effect of carotid sinus pressure on 50 predominantly male subjects, 28 of whom had a resultant fall in systolic blood pressure at a mean of 23% of the baseline level. As the fall in blood pressure was independent of cardiac slowing, Koch assumed that a depressor vasomotor reflex was in operation (13). Mehrmann confirmed these observations, noting a particularly marked fall in blood pressure in patients with arteriosclerotic disease (18), as did Mandelstamm and Lifschitz (19), who also demonstrated a particularly marked fall in blood pressure in subjects with hypertension as well as arteriosclerosis.

Mandelstamn and Lifschitz were also the first to associate the more pronounced hemodynamic consequences of carotid sinus stimulation in relation to advancing age; the 103 retired Russian workers studied had an average fall in systolic blood pressure of 37 mmHg with carotid sinus pressure, while 106 healthy young soldiers had only a 5-mmHg vasodepressor response (19). They also noted that the degree of heart rate slowing did not necessarily correlate with that of the fall in blood pressure and that the fall in heart rate occurred earlier and lasted for a much shorter time than the blood pressure fall (19). Moreover, Mandelstamm and Lifschitz (19) were the first to emphasize the need for uniformity in the technique of carotid sinus pressure in man. The patient should lie supine, with the head elevated, just overhanging a support and turned slightly to one side, the sinus being located at the angle of the jaw and at the upper border of the thyroid cartilage (19).

The first case report of syncope and pre-syncope caused by a pathological carotid sinus reflex was published by Roskam in 1930, along with the original use of the term “hypersensitivity” (“*hyperreflectivité*”) (20). The 53-year-old man described had recurrent syncope first elicited by stretching of the skin while shaving. During clinical examination, the compression of the carotid sinus caused more than 15 s of asystole with loss of consciousness and “convulsions,” as graphically described by Roskam: “…pendant cette syncope que se prologea plus de quinze secondes apres la fin de l'attouchement, j'auscultai avec la plus grande attention la region precordiale: silence absolu. Finalement, survinrent des convulsions epileptiformes generalisees. Puis brusquement, le coeur se remit a battre sur un rythme accelere, a 120 pulsations environ a la minute, des extrasystoles venant frequemment entrecouper la succession precipitee des systoles regulieres” (20)^*^. Repeated light carotid sinus pressure resulted in 16 s of asystole, again with syncope and convulsions. The patient was treated successfully with atropine and remained symptom-free at follow-up (20).

[^**^ “…as syncope occurred for more than 15 sec. following discontinuation of pressure, I auscultated attentively over the praecordium: absolute silence. Finally, generalised epileptiform convulsions ensued. Then, abruptly, the heart began to beat with an accelerated rhythm, at around 120 beats per minute, with initial frequent extrasystoles interrupting the succession to normal sinus rhythm”]

The millennia-long foundations had therefore been laid for Soma Weiss and James Baker's landmark case series in carotid sinus hypersensitivity (CSH) published later in 1933, describing “the carotid sinus reflex in health and disease” and “its role in the causation of fainting and convulsions” (21). Fifteen subjects with CSH, all with symptom reproduction during carotid sinus pressure of variable degrees and duration, were described in detail, with the division of responses to carotid stimulation designated as “vagal” where marked bradycardia or asystole occurred, “depressor” where arterial pressure fell independently of cardiac slowing, and “cerebral” where syncope occurred with no hemodynamic changes, although this last type soon proved secondary to cerebral anterior circulatory compromise caused by carotid artery obliteration during carotid sinus massage (CSM) in the presence of hemodynamically significant contralateral carotid stenosis (22, 23).

**Figure 4:**
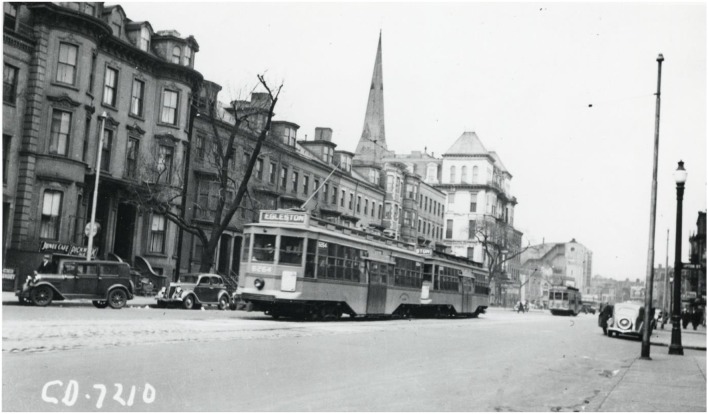
1930s Boston streetcar. A two-car train of center-entrance streetcars on Tremont Street at Upton Street (just north of Dartmouth Street), probably in the 1930s. From City of Boston Archives, West Roxbury, USA, via Wikimedia Commons (https://www.commons.wikimedia.org/wiki/File:Streetcars_on_Tremont_Street_opposite_Upton_Street,_1930s.jpg).

While the lack of standardization of carotid sinus stimulation, *ad hoc* subject selection, and absence of diagnostic definitions hamper Weiss and Baker's original paper, their contribution, in terms of drawing attention to the pathologic role of the carotid sinus and making some sense of the presentation, natural history, and management of the condition, is unique. One of the case reports presented in the paper was on a 65-year-old Boston streetcar driver (Figure 4) with fainting and dizziness upon turning his head from side to side to look out for traffic, which has passed into medical folklore. The patient was found to have reproducible CSH, and the characteristic hemodynamic responses were later reproduced with the celluloid high collar he used for work, with all symptoms resolving with the use of a soft collar! (21) As Mehrmann (18), Mandelstamm and Lifschitz (19), and Nathanson (24) had noted, the hypersensitive response was more common in patients with arteriosclerotic disease, with all but one of Weiss and Baker's subjects being so affected (21). They also noted that “pressure on the sinus regularly brought on fainting more quickly when the patient was standing than when he was lying down” (21), a finding confirmed and reinforced since (25).

The stage was thus set for further clinical exploration of Weiss and Baker's “syndrome of dizziness, fainting and convulsions due to a hyperactive carotid sinus reflex” (21). The management of carotid sinus syndrome (CSS; CSH in response to CSM culpably associated with symptoms of syncope, dizziness, drop attacks, or unexplained falls vs. CSH in isolation, which is not associated with such symptoms) was initially with vagolytics or carotid sinus denervation (26) until the first permanent pacemaker was implanted for CSS almost half a century ago (27). This treatment strategy for the management of CSS' cardioinhibitory and mixed subtypes has continued ever since and is supported by international consensus guidance to this day (28–32). However, a growing body of evidence from a number of epidemiologic, experimental, and observational studies has questioned whether CSS is a disease state in need of treatment or a coincidental finding (33–36). Alongside this, international systematic reviews and meta-analyses consistently and inconveniently demonstrate the lack of high-quality evidence for permanent pacing in the management of CSS (30, 32).

So how to disentangle fact from fiction (or at best supposition) in the question of whether we should ever pace for CSS? Before trying to establish a final answer, it is instructive to decide whether there is a disease process at work, as without a disease (or at minimum, a symptomatic deviation from normal function), there is no case for treatment. While the Oxford Dictionary definition of disease as “a disorder of structure or function in a human, animal, or plant, especially one that produces specific symptoms or that affects a specific location and is not simply a direct result of physical injury” (37) may seem to fit CSS, a more practical and informative definition might flow logically from the following questions:

1. Is there a pathophysiologic rationale for the disease (in this case CSS)?
2. Is there a good diagnostic test that will identify it reliably?
3. Is there a convincingly evidence-based treatment for the disease?

In the remainder of this paper, I will discuss each of these in the context of the CSS subtypes for which permanent pacing is indicated (cardioinhibitory and mixed) before attempting to synthesize the answers into a coherent response to its title. Vasodepressor CSS is not the subject of this review.

## Is There a Pathophysiologic Rationale for Carotid Sinus Syndrome?

**Figure 5:**
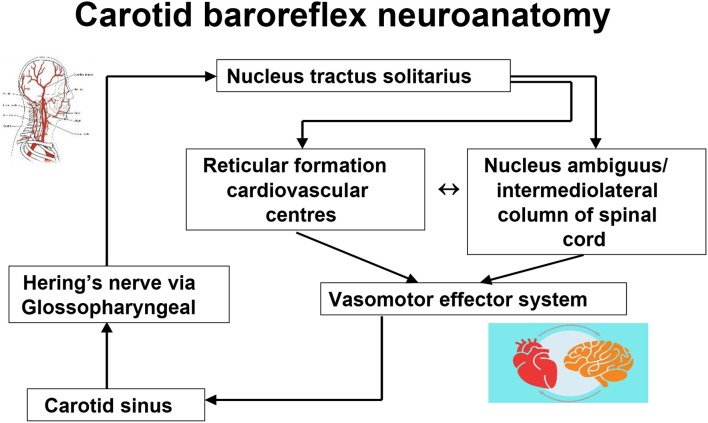
Carotid baroreflex neuroanatomy.

It is evident from the historical overview above that, first, the stimulation of the carotid sinus provokes exaggerated heart rate and blood pressure changes in normal humans (carotid sinus hypersensitivity), and second, that in some individuals, stimulation through the carotid sinus pressure or massage can provoke syncope (carotid sinus syndrome). What is less evident is what might cause the conversion of the asymptomatic state to the symptomatic state. The basic functional neuroanatomy of the carotid sinus reflex has an afferent component from the sinus *via* neuronal projections to the brainstem [in particular, the nucleus tractus solitarius (38, 39)] via Hering's nerve and the glossopharyngeal nerve, while the efferent expressions of CSH are mediated by the vagus nerve in cardioinhibitory carotid sinus hypersensitivity (40–42) and by sympathetic withdrawal, with subsequent vasodilatation and arterial hypotension in mixed carotid hypersensitivity and vasodepressor carotid hypersensitivity (Figure 5) (42–45). Why the exaggerated hemodynamic responses are triggered is not understood. Certainly the carotid sinus and its projections are unlikely culprits as the histology of the intima and the nerve terminals in CSS is essentially normal (38, 46), and both the vasodepressor and the cardioinhibitory effects of CSM continue despite the termination of carotid stimulation (and carotid sinus neural output) (40, 43), Furthermore, denervation of the carotid sinus is not always a successful intervention in the management of CSH (47, 48). Although the sinus itself may be an unlikely primary source of the hypersensitive response, Tea et al. (49) and later Blanc et al. (50), working in the same laboratory, found a powerful (and unexpected) association between electromyographically demonstrated sternocleidomastoid muscle denervation and CSH during CSM (49, 50). The authors suggest that the chronic loss of innervation of the sternocleidomastoid muscles may cause an increased sensitivity of the baroreflex arc and hence CSH, although the link is tenous (49). However, causality in the opposite direction must be considered—there is no evidence to refute the possibility of sternocleidomastoid denervation as a *consequence* of CSH. This important work has never been replicated or explored further.

One study showed hyperphosphorylated tau accumulation baroreflex-associated neurons in a controlled neuroanatomical study of 12 patients with CSH compared to 14 controls (51), so there is some evidence to support a central neuropathologic culprit in CSH, although this finding has not been examined elsewhere. The efferent limb of the carotid baroreflex arc appears to be intact given the exaggerated vasodepression and normal bradycardic response to muscarinic stimulation with edrophonium seen in CSH (5). By exclusion, a central brain-stem-level abnormality in the modulation of central baroreflex gain is therefore likely and indeed was suggested three decades ago (52), although interestingly Tea et al.'s study found no abnormalities of the central neurophysiological parameters in subjects with CSH (49). One hypothesis suggests that central alpha-2 adrenoceptor upregulation provides the substrate for this baroreflex gain (53), with reduced arterial compliance secondary to carotid arteriosclerosis associated with ageing, hypertension (54), or atheroma resulting in diminished mechano- and baroreceptor stimulation and thus a decrease in afferent neural traffic to the brain stem, resulting in the upregulation of medullary alpha-2 adrenoceptors, which are known to regulate negative feedback hypotensive and bradycardic responses (55).

**Figure 6:**
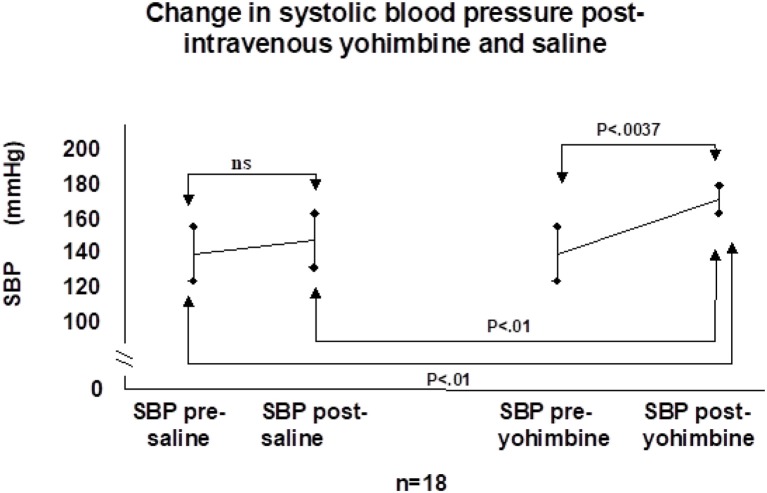
Change in systolic blood pressure post-intravenous yohimbine and saline. SBP, systolic blood pressure; ns, not significant [from Parry et al. (56)].

This “physiologic” denervation hypersensitivity then causes the overshoot bradycardia and hypotension following carotid sinus stimulation that is clinical CSH (53). We tested this hypothesis with a double-blind, placebo-controlled cross-over study of the centrally active alpha-2 adrenoceptor antagonist yohimbine administered during CSM in patients with documented CSS (56). If the alpha-2 adrenoceptor hypothesis was true, the hemodynamic responses to yohimbine should be markedly attenuated—this was not the case (Figure 6) (56). More recently, 10 older adults with CSH had higher arterial stiffness and reduced arterial baroreflex sensitivity compared to those without, further providing no evidence to support the upregulation of the arterial baroreflex in patients with CSH (57).

There is thus a small but inconclusive evidence base to suggest neuroanatomical abnormalities as the underpinnings of CSS. What about a more functional disorder analogous to psychiatric disease in the absence of overt brain pathology? One possibility is disordered cerebral autoregulation, a candidate catalyst for the conversion of asymptomatic CSH to CSS, with linked studies by Leftheriotis et al. (58, 59) showing that hypotension secondary to CSM caused the delayed onset of transcranial Doppler ultrasonographically (TCD)-measured cerebral autoregulation which was more prominent with increasing asystole duration. Dual-chamber pacing ameliorated the response. However, the studies were small, uncontrolled, and provided no method of distinguishing cerebral autoregulatory derangements specific to carotid sinus stimulation from the effects of profound arterial hypotension, during which cerebral autoregulation falters and fails (60). We sought to overcome these limitations through a comparison of changes in cerebral autoregulation (as measured by TCD) in response to controlled lower body negative pressure-induced hypotension in subjects with CSS, those with asymptomatic CSH, and in healthy controls in a series of studies (61, 62). In both studies, we found evidence of deranged cerebral autoregulation, in the first particularly through differences in cerebrovascular resistance and in the second in cerebral blood flow velocity between patients with CSS and controls (61, 62). However, the findings have not been replicated elsewhere and suffer from the small sample sizes as well as the many limitations of the TCD method of estimating cerebral autoregulation. Further work is needed before definitive conclusions can be drawn.

If cerebral autoregulation abnormalities are the ultimate expression of the cause of symptoms in CSS, a strong candidate for the mediating mechanism would be the same underlying process, autonomic dysfunction. Morley and Sutton found abnormal baroreflex sensitivity in CSS and sick sinus syndromes as measured by the phenylephrine pressor test (63). Almost three decades later, we studied baroreflex sensitivity and heart rate variability in 22 patients with CSS, 18 with CSH, and 14 normal controls only to find that both CSS and CSH patients had increased resting sympathetic activity and baroreflex sensitivity compared to controls (35). Whether this reflects a generalized mild autonomic dysfunction associated with aging or a pathologic state remains unknown. We further explored the autonomic hypothesis through meta-iodo benzyl guanidine scanning of cardiac sympathetic activity in patients with CSS, patients with CSH, and asymptomatic controls (64). Cardiac sympathetic neuronal activity was increased in patients with CSS, but not in the other two groups (64), adding more weight to the suggestion that CSS is a clinical manifestation of autonomic dysregulation in older individuals.

## Is There a Good Diagnostic Test That Will Identify Carotid Sinus Syndrome Reliably?

Consensus guidelines state that the current standard diagnostic test for CSS is 10 s of bilateral, sequential, and longitudinal CSM, right then left (as the hypersensitive response is more likely to occur on the right), during electrocardiographic and (preferably beat-to-beat) blood pressure monitoring (29, 31, 65). The process should be repeated in the upright position in order to avoid missing up to a third of cases (25), with diagnosis of CSS confirmed by the presence of prolonged asystole with reproduction of usual symptoms (29, 31, 65, 66). The duration of asystole deemed as diagnostic was unspecified early on, 3 s or more for half a century, but now more than 6 s, following recent arguments detailing the duration of cardiac pause needed to induce loss of consciousness (67). However, this consensus certainly masks an absolute dearth of rigorous experimental effort to support it, alongside the fact that the distinction between carotid sinus pressure (Weiss and Baker's original method) and massage gained traction in the late 1960s (68) and became commonplace only in the 1980s (52, 63, 69). Moreover, the duration of massage similarly has little basis in scientific methodology, with durations of up to 30 s of pressure or massage for much of the 1930s to 1980s (68, 70), and 5 s in many laboratories (66) and recent American syncope guidelines (28). Added to this, despite confidently quoted anatomical landmarks (65, 66), the carotid sinus' position can vary considerably, with the estimated location missing the actual location by up to 1.5 cm (71), and post-mortem evaluation shows a high variation in sinus location, with an asymmetric location in 34% (72). The implications for further diagnostic ambiguity are clear, particularly regarding false-negative tests.

## Is There a Convincingly Evidence-Based Treatment for the Disease?

The short answer to this question is no, if the standard of evidence required is that of the randomized controlled trial (RCT). Two recent high-quality reviews of pacing intervention in CSS, where syncope is the presenting complaint, found no high-quality evidence to support pacing as a treatment of choice (30, 32) despite the consensus guideline strength of recommendation being set at IIa or IIb (28, 29, 31). However, this masks the dearth of RCTs on which to base gold-standard treatment recommendations and the wealth of observational data supporting pacing as an effective intervention (26, 29, 73–75). Such data come with considerable methodologic baggage and innumerable biases and need further investigation. There is little further clarity where CSH has been associated with unexplained falls, although not according to the method of symptoms (i.e., in the absence of symptom reproduction during positive CSM). Several studies have examined the role of pacing in this context (76–78). The SAFE PACE study showed a significant reduction in fall rates in those paced vs. those without pacing intervention in CSH fallers, although the magnitude of intervention (surgical procedure vs. no intervention) makes interpretation more difficult (76). The latter SAFE PACE 2 study, with a more rigorous study design (pacing vs. implantable loop recorder, so both arms had device intervention), showed no such benefits (77). Similarly, the only randomized, double-blind, placebo-controlled pacing intervention study in this area (indeed in any CSS study) showed no reduction in fall rates with pacing, although the study was marginally underpowered (78). The mechanism of causality, and whether pacing is effective or not in unexplained falls, is thus as unclear as in syncope.

## Should We Ever Pace for Carotid Sinus Syndrome?

“*The truth is rarely pure, and never simple”*

(Oscar Wilde, The Importance of Being Earnest, Act I, 1895)

Truth, in the sphere of day-to-day existence as much as in classic 19th century comedy, is seldom absolute. As the reader will be aware from the discussion so far, Wilde's pithy observation on the nature of truth has a particular resonance in attempting to answer the question posed by the title of this paper. Our putative disease, CSS, on balance from the small number studies and patients involved in trying to understand the physiologic bedrock of this elusive condition, appears to have some basis in autonomic dysfunction. What is less apparent is whether this represents a disease in need of management (at least in the sense of the word as here defined) or whether this is part of the autonomic spectrum of normal aging. On balance, the former seems more likely, although a definitive answer is not possible from the data so far available, with the distinction between CSH and CSS proving particularly difficult from a pathophysiologic perspective. To further make the waters muddy, there is considerable observational evidence to suggest that there are large numbers of older people who have CSH in the absence of symptoms. Kerr et al. systematically evaluated a random sample of community-dwelling elders and found that 39% had CSH overall, with a cardioinhibitory response in 24%—in the absence of any culpable symptoms (33). Older studies with thousands of subjects found that 4–41% had CSH (24, 79, 80), with a particularly high prevalence in those with coronary artery disease (81) with or without culpable symptoms.

More troublesome is the changing face of the technique and criteria used to diagnose this apparent disease, morphing from “pressure” of up to 30 s to the current 10 s of longitudinal massage over the course of the last eight or nine decades. Current guidance, on very sound physiologic principles, defines the cutoff for CSS diagnosis as 6 s of asystole, ignoring the troublesome fact that many of the intervention studies since the 1980s used the 3 s criterion (26, 28, 30, 73) to establish the diagnosis. Additionally, current consensus guidelines on pacing in CSS base their entire (fairly strong) recommendation on such observational studies with apparently successful pacing in patients with CSS—diagnosed using the 3 s criterion, many with 5 s massage. So, while pacing may reduce syncope burden in CSS, there is little high-quality evidence supporting that it does so.

The answer to the question posed therefore is … probably. There is some evidence of disordered physiology in need of a remedy to treat the symptoms, although the test to diagnose is not a good one and the evidence supporting the intervention is arguably weaker than the strength of the recommendations for its use. Without a doubt, much further work is needed, with more detailed work on pathophysiology to guide treatment strategies, a better diagnostic test, and more clear phenotyping of symptom presentation that then aligns with potential pacing intervention. In addition, given the differences in test performance and interpretation as well as evidence level recommendations, it would be useful to develop world-wide consensus on the diagnosis and management of CSS (Box 1). Newer potential treatments for CSS' sister, neurally mediated condition vasovagal syncope, may offer additional therapeutic benefit and need evaluation, for example, autonomic modulation using parasympathetic cardiac ganglionic plexi ablation (82) and drug treatment with the norepinephrine transporter inhibitor atomoxetine (83).

Box 1 **Pacing in Carotid Sinus Syndrome: European Society of Cardiology (29) and American College of Cardiology/American Heart Association/Heart Rhythm Society (28) Consensus.**

***Patient characteristics***

Age 40 or over, presenting with syncope^*^

No recent stroke, transient ischaemic attack or myorcardial infarction and no significant carotid stenosis (>70% in ESC guidance (29), though neither advocates routine carotid Doppler study screening prior to carotid sinus massage)

***Carotid sinus massage and interpretation of test result***

Locate carotid sinus as point of maximal carotid pulsation between the angle of the jaw and the cricoid cartilage

Ten seconds^±^ bilateral, sequential, longitudinal carotid sinus massage, right then left, supine then upright with continuous ECG and beat-to-beat blood pressure monitoring

***Positive test***

Symptom reproduction during more than six seconds^±^ asystole

- Cardioinhibitory CSS: asystole without significant vasodepression (i.e., 50 mmHg fall in systolic blood pressure)
- Mixed CSS: asystole with significant vasodepressor response.^§^

***Management***

Modification of culpable medication where feasible

Dual chamber pacemaker implantation may be indicated for cardioinhibitory or mixed CSS sub-typesGuidance

^*^While neither guideline expressly suggests massage in patients with unexplained fall or drop attacks that are likely to be syncopal, it is our centre's practice to do so in such individuals given the discussion above

^±^Five seconds massage, and more than three seconds asystole for test positivity in North American guidelines (28)

^§^The European guidelines suggest repeated CSM with intravenous atropine injection to distinguish predominant cardioinhibition from vasodepression in mixed sub-type CSS (29) in order to characterise more accurately the relative contributions of asystole and non-asystole related hypotension

^§^Levels of evidence for pacing intervention are IIa in North American (28) and IIb in European guidelines (29)

If a slavish adherence to a gold-standard evidence base is to be the sole guiding principle, pacing cannot be recommended for CSS. However, in the real world of patient care where clinical experience chimes with the weight of history, while further evidence is rigorously sought, it is not unreasonable to follow imperfect but sensible consensus guidance until an unambiguous verdict is reached (Box 1).

## Author Contributions

The author confirms being the sole contributor of this work and has approved it for publication.

## Conflict of Interest

The author declares that the research was conducted in the absence of any commercial or financial relationships that could be construed as a potential conflict of interest.
